# The Effect of Botulinum Toxin Type A on Expression Profiling of Long Noncoding RNAs in Human Dermal Fibroblasts

**DOI:** 10.1155/2017/2957941

**Published:** 2017-02-07

**Authors:** Ying-ying Miao, Juan Liu, Jie Zhu, Yan-ling Tao, Jia-an Zhang, Dan Luo, Bing-rong Zhou

**Affiliations:** ^1^Department of Dermatology, The First Affiliated Hospital of Nanjing Medical University, Nanjing 210029, China; ^2^Department of Dermatology, The First Affiliated Hospital of Nanjing University of TCM, Nanjing, Jiangsu 210029, China

## Abstract

*Objective*. This study was aimed at analyzing the expressions of long noncoding RNAs (lncRNAs) in Botulinum Toxin Type A (BoNTA) treated human dermal fibroblasts (HDFs) in vitro.* Methods*. We used RNA sequencing to characterize the lncRNAs and mRNAs transcriptome in the control and BoNTA treated group, in conjunction with application of GO (gene ontology) analysis and KEGG (kyoto encyclopedia of genes and genomes) analysis to delineate the alterations in gene expression. We also obtained quantitative real time polymerase chain reaction (qRT-PCR) to confirm some differentially expressed genes.* Results*. Numerous differentially expressed genes were observed by microarrays between the two groups. qRT-PCR confirmed the changes of six lncRNAs (RP11-517C16.2-001, FR271872, LOC283352, RP11-401E9.3, FGFR3P, and XXbac-BPG16N22.5) and nine mRNAs (NOS2, C13orf15, FOS, FCN2, SPINT1, PLAC8, BIRC5, NOS2, and COL19A1). Farther studies indicated that the downregulating effect of BoNTA on the expression of FGFR3P was time-related and the dosage of BoNTA at a range from 2.5 U/10^6^ cells to 7.5 U/10^6^ cells increased the expression of FGFR3P and COL19A1 in HDFs as well.* Conclusion*. The expression profiling of lncRNAs was visibly changed in BoNTA treated HDFs. Further studies should focus on several lncRNAs to investigate their functions in BoNTA treated HDFs and the underlying mechanisms.

## 1. Introduction

Botulinum Toxin Type A (BoNTA) is the most effective one among the seven neurotoxins secreted by* Clostridium botulinum* [1]. BoNTA causes muscle relaxation and this concept nowadays is widely being used in the cosmetic treatment of wrinkles [2, 3]. In 2005 and 2008, some researchers found a face-lifting effect after intradermal injection of BoNTA to the mid and lower face [4, 5]. However, some other researchers reported that needle pricks themselves without BoNTA can make the skin become smoother as well [6]. In order to find out whether BoNTA can affect the human dermal fibroblasts (HDFs) directly, in 2012, Oh et al. studied the in vitro effects of BoNTA on normal HDFs and found that BoNTA has a notable effect in increasing the level of collagen production and downregulating its degradation [7]. Collagen is the most abundant basic element of fibrous components in the dermis and is responsible for maintaining the structural integrity of the skin by joining cells together and to the extracellular matrix (ECM) [8, 9]. These studies not only showed the positive effects of BoNTA on HDFs for remodeling skin but also implied the importance of HDFs. In 2016, Zhu et al. proved that topical BoNTA application could enhance the rejuvenation effect of fractional CO_2_ laser, further indicating that BoNTA can refine skin texture via improving the activity of HDFs [10]. But until now, the molecular mechanisms through which BoNTA could affect HDFs are still not completely understood.

Long noncoding RNAs (lncRNAs) are a group of noncoding RNA transcripts longer than 200 nucleotides which cannot encode proteins [11]. In comparison with protein-coding genes, lncRNAs have limited coding potential and show little evolutionary conservation in sequence. Furthermore, some researchers have detected that lncRNAs expression is more tissue specific and at apparently lower levels [12]. LncRNAs, which were previously thought to be transcriptional “noise,” are now proved to have some functions by regulating gene expression at the epigenetic, transcriptional, and posttranscriptional levels and participating in some biologic functions, such as genomic imprinting, chromosome modification, intranuclear transport, transcriptional activation, and interference [13]. Therefore, the understanding of cellular processes in physiological conditions will not be complete without analyzing the contributions made by lncRNAs. Until now, no information is available regarding the effect of BoNTA on expression profiling of lncRNAs in HDFs.

In this study, we investigated on lncRNA expression signature together with messenger RNA (mRNA) expression profile in BoNTA treated HDFs and confirmed the changing of some differentially expressed lncRNAs and mRNA using qRT-PCR. In conjunction, we also conducted functional analysis using Gene Ontology (GO) analysis and pathway analysis, in which genes are mapped to Kyoto Encyclopedia of Genes and Genomes (KEGG) pathways.

## 2. Materials and Methods

### 2.1. Cell Separation and Culture

Normal human skin samples were obtained from the prepuce of young healthy individuals in accordance with the ethics committee approval process of The First Affiliated Hospital of Nanjing Medical University (Nanjing, China). The acquirement of HDFs can be divided into two procedures. Initially dispase enzyme was used to separate the dermis and epidermis, and then collagenase enzyme was used to extract the HDFs. HDFs were grown in Dulbecco's modified Eagle medium (DMEM) with 1% penicillin-streptomycin and 10% fetal bovine serum in an environment of 5% CO_2_ at 37°C. The cells used in our study were from passages 8–11.

### 2.2. Group Divisions and Botulinum Toxin Type A (BoNTA) Treatment

In order to study differentially expressed lncRNAs and mRNAs, we separated the cells into two groups, control group and BoNTA group: (1) control group: HDFs were grown in DMEM with 1% penicillin-streptomycin and 10% fetal bovine serum for 5 days and then serum-starved for 4 days, without receiving BoNTA treatment; (2) BoNTA group (48 h): HDFs were grown in DMEM with 1% penicillin-streptomycin and 10% fetal bovine serum for 5 days, serum-starved for 2 days, and then were grown in serum-free DMEM with BoNTA at a dose of 5 U/10^6^ cells for 2 days.

In order to determine whether the changes of RNAs expression in BoNTA treated HDFs were time or dosage dependent, the cells were divided into 4 groups: (1) BoNTA group (24 h): HDFs were grown in DMEM with 1% penicillin-streptomycin and 10% fetal bovine serum for 5 days, serum-starved for 2 days, and then were grown in serum-free DMEM with BoNTA at a dose of 5 U/10^6^ cells for 24 h; (2) BoNTA group (72 h): HDFs were grown in DMEM with 1% penicillin-streptomycin and 10% fetal bovine serum for 5 days, serum-starved for 2 days, and then were grown in serum-free DMEM with BoNTA at a dose of 5 U/10^6^ cells for 72 days; (3) BoNTA group (48 h 2.5 U): HDFs were grown in DMEM with 1% penicillin-streptomycin and 10% fetal bovine serum for 5 days, serum-starved for 48 h, and then were grown in serum-free DMEM with BoNTA at a dose of 2.5 U/10^6^ cells for 48 h; (4) BoNTA group (48 h 7.5 U): HDFs were grown in DMEM with 1% penicillin-streptomycin and 10% fetal bovine serum for 5 days, serum-starved for 2 days, and then were grown in serum-free DMEM with BoNTA at a dose of 7.5 U/10^6^ cells for 48 h.

All groups were rinsed with Phosphate Buffer Solution (PBS) and the medium was changed every day, except during BoNTA treatment. Each method of detection consists of these six groups of cell culture, containing about 1 × 10^7^ cells in each group, and there were at least 3 samples in each group. The BoNTA used in this study was manufactured by Lanzhou Institute of Biological Products Co., Ltd., Lanzhou, China.

### 2.3. Isolation of RNA and Preparation of Array Hybridization

The RNA extraction was conducted using the TRIZOL reagent and then was dissolved in RNase-free water. The purified labeled genomic DNA was used for quantification and the RNA quantity was decided spectrophotometrically as A260/A280 ratio (1.9–2.1 were obtained). The collected RNAs were stored at −70°C for microarray analysis and qRT-PCR.

### 2.4. Microarray Analysis of lncRNAs and mRNAs Expression

The Agilent Human lncRNA (8*∗*60 K) arrays were designed in this experiment for measuring the expression profiles of lncRNAs and mRNAs. The lncRNA sequences were acquired from the following databases: NCBI-RefSeq, NONCODE v4, Ensembl, broad lincRNA, and frnadb v3.4. We conducted the sample labeling, microarray hybridization, and washing according to the manufacturer's proposals. Briefly speaking, we initially transcribed the total RNA to double stranded cDNA, synthesized them into cRNA, and labeled them with Cyanine-3-CTP and then hybridized the labeled cRNAs onto the microarray. We applied the Agilent Scanner G2505C (Agilent Technologies) to scan the arrays after washing.

### 2.5. Quantitative Reverse-Transcription Polymerase Chain Reaction (qRT-PCR)

In order to confirm the results obtained from microarray, we applied qRT-PCR to remeasure the abundance of differentially expressed lncRNAs and mRNAs selected from microarray analysis. RNAs in the medium were determined based on the protocol of KeyGen Biotech Co., Ltd., Nanjing, Jiangsu, China. In brief, we applied TRIzol to extract the total RNAs from HDFs and then synthesized the cDNAs from the separated RNA using SuperScript III Reverse Transcriptase (KeyGen, China). QRT-PCR was performed on ABI Prism 7700 Sequence Detector (Applied Biosystems). The reactions were performed at 9°C for 10 min, then at 40 cycles at 95°C for 15 s, and followed by at 60°C for 30 s. The 2 (−ΔΔCt) methods were used to evaluate relative quantification of lncRNAs and mRNAs expression. Primer sequences of lncRNAs and mRNAs for qRT-PCR are listed in Table 1. The results were expressed as mean ± SD (standard deviation) of each independent experiment.

### 2.6. Data Analysis

We applied the Feature Extraction software (version 10.7.1.1, Agilent Technologies) to analyze array images to get raw data and used GeneSpring (version 13.1, Agilent Technologies) to complete the fundamental analysis with the raw data. First of all, the raw data was normalized with the quantile algorithm. The probes which have signed with* P* (*p* value ≤0.05 is recommended) were selected for further data analysis. Deferentially expressed lncRNAs and protein-coding RNAs were then identified through fold change. The critical value set for up- and downregulated genes was a fold change ≥ 2.0. Afterwards, GO analysis and KEGG analysis were applied to depict alterations in the gene expression. SPSS 17.0 software (SPSS Inc., Chicago, IL, United States) was used to analyze the statistical data. The differences in RNA expression between the two groups and more than three groups were analyzed using the Student's* t*-test and one-way ANOVA, separately. *p* < 0.05 was considered to indicate a statistically significant difference.

## 3. Results

### 3.1. Different Expression Profiles of lncRNAs and mRNAs in Control Group and BoNTA Treated Group

**Figure 1:**
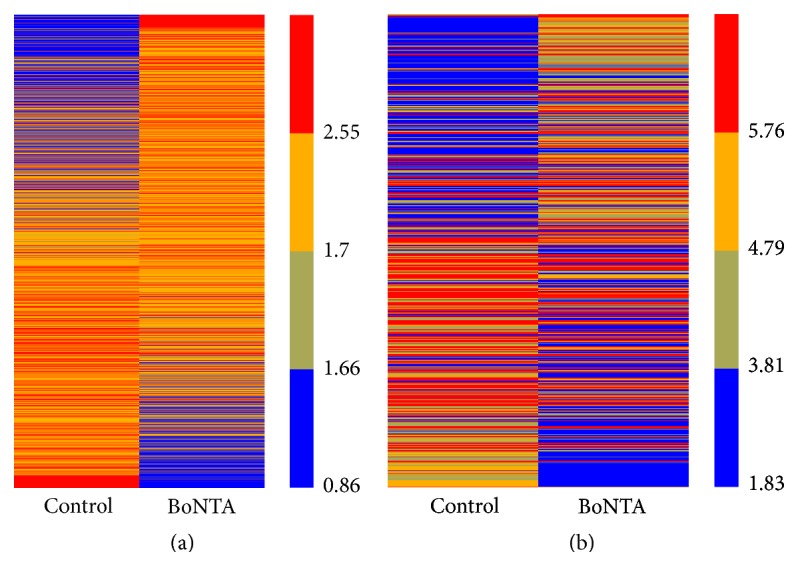
Heat maps presentation of the expression profiling of mRNAs (a) and lncRNAs (b) in different groups of HDFs. Red and blue represent high and low relative expressions, respectively. Control indicates the normal HDFs and BoNTA indicates the HDFs treated by BoNTA (5 U/10^6^ cells 48 h).

Data analysis showed 2124 differentially expressed lncRNAs and 638 differentially expressed mRNAs of the cells in BoNTA (48 h 5 U) treated group (fold change > 2) compared with control group. Hierarchical clustering heat-map showed the expression ratios of mRNAs (Figure 1(a)) and lncRNAs (Figure 1(b)) between the two groups. Of the 2124 differentially expressed lncRNAs, 1122 were upregulated and 1002 were downregulated. Of the 638 differentially expressed mRNAs, 303 were upregulated and 335 were downregulated. The distinctly expressed lncRNAs are listed in Table 2 (fold change > 5) and the distinctly expressed mRNAs are summarized in Table 3 (fold change > 3).

### 3.2. QRT-PCR Analysis of lncRNAs and mRNAs Expression

**Figure 2:**
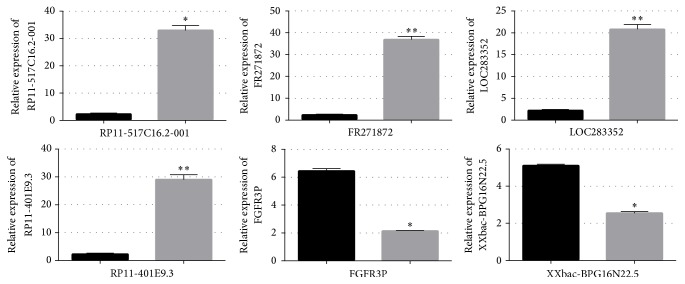
The differential expression level of long noncoding RNAs (lncRNAs) between control and BoNTA (5 U/10^6^ cells 48 h) treated groups was validated by qRT-PCR. Six lncRNAs (RP11-517C16.2-001, FR271872, LOC283352, RP11-401E9.3, FGFR3P, and XXbac-BPG16N22.5) were studied using glyceraldehyde-3-phosphate dehydrogenase (GAPDH) as an internal control. The heights of the columns in the chart represent the fold changes. Data are the mean ± SEM (*n* = 6). *p* < 0.05 was considered to indicate a statistically significant difference compared with control HDFs. ^*∗*^*p* < 0.05 and ^*∗∗*^*p* < 0.01.

QRT-PCR analysis indicated the transcription of the selected lncRNAs: RP11-517C16.2-001, FR271872, LOC283352, RP11-401E9.3, FGFR3P, XXbac-BPG16N22.5, and mRNAs: collagen 19a1 (COL19A1), nitric oxide synthase 2 (NOS2), chromosome 13 open reading frame 15 (C13orf15), FBJ murine osteosarcoma viral oncogene homolog (FOS), ficolin (collagen/fibrinogen domain containing lectin) 2 (hucolin) (FCN2), serine peptidase inhibitor, Kunitz type 1 (SPINT1), placenta-specific 8 (PLAC8), E2F transcription factor 1 (E2F1), and baculoviral IAP repeat containing 5 (BIRC5), perfectly correlated to microarray results. The qRT-PCR analysis revealed that the expression level of RP11-517C16.2-001, FR271872, LOC283352, and RP11-401E9.3 in BoNTA (5 U 48 h) treated groups was upregulated to 33.479-, 39.519-, 19.713-, 26.362-, and 29.293-fold separately (Figure 2), and the expression level of FGFR3P and XXbac-BPG16N22.5 was downregulated to 2.139- and 2.554-fold, respectively (Figure 2).

**Figure 3:**
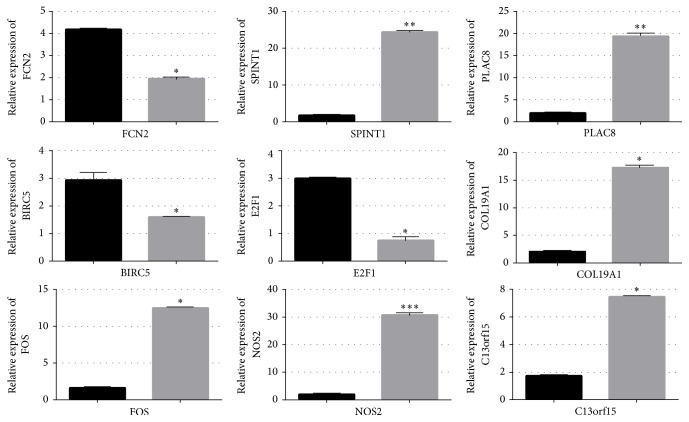
The differential expression levels of message RNAs (mRNAs) between control and BoNTA (5 U/10^6^ cells 48 h) treated groups of microarray results were validated by qRT-PCR. Nine mRNAs (FCN2, SPINT1, PLAC, BIRC5, E2F1, COL19A1, FOS, NOS2, and C13orf15) were studied using glyceraldehyde-3-phosphate dehydrogenase (GAPDH) as an internal control. The heights of the columns in the chart represent the fold changes. Data are the mean ± SEM (*n* = 6). *p* < 0.05 was considered to indicate a statistically significant difference compared with control HDFs. ^*∗*^*p* < 0.05, ^*∗∗*^*p* < 0.01, and ^*∗∗∗*^*p* < 0.001.

For the mRNAs, in comparison with the control group, the expression level of COL19A1, NOS2, C13orf15, FOS, SPINT1, and PLAC8 was upregulated to 10.331-, 31.374-, 7.534-, 12.573-, 24.758-, and 19.885-fold in BoNTA (5 U 24 h) treated groups, respectively (Figure 3), and the expression level of FCN2, BIRC5, and E2F1 was downregulated to 1.890-, 0.923-, 0.709-fold separately (Figure 3).

### 3.3. GO and Pathway Analysis of Deferentially Expressed RNAs

**Figure 4:**
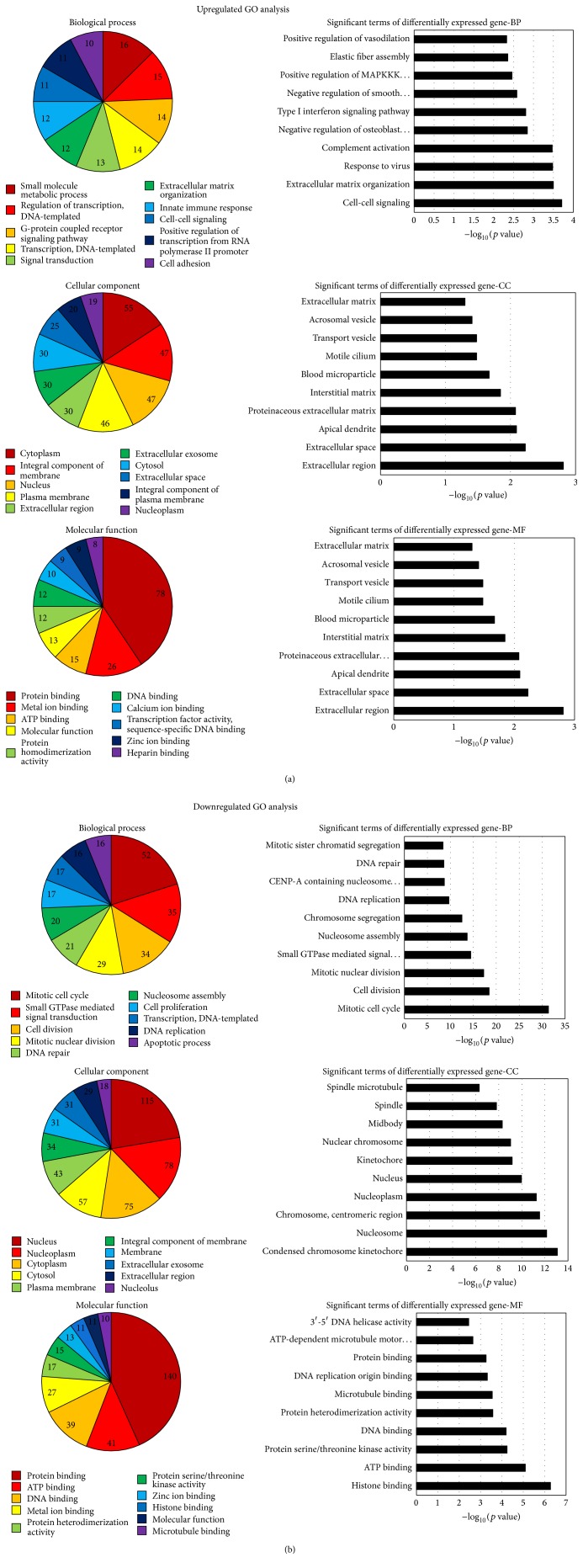
Bioinformatic analysis of the differentially expressed genes. The* p* value denotes the significance of GO terms enrichment in the differentially expressed genes. The lower the* p* value, the more significant the GO term (*p* value ≤ 0.05 is recommended). We can choose the target genes for further study based on the combination of the analysis provided by GO and the biologic significance.

The upregulated genes were involved in 898 biological processes, 171 cellular components, and 236 molecular functions separately. The response to cell-cell signaling (−log_10_ (*p* value) = 3.7114) was the most significant term among the biological process category. The extracellular region (−log_10_ (*p* value) = 2.8128) was the most represented GO term in the cellular component category. Heparin binding (−log_10_ (*p* value) = 3.3714) was the most highly represented term within the molecular component category. The downregulated genes were involved in 880 biological processes, 179 cellular components, and 240 molecular functions, respectively. The most significant term was the response to mitotic cell cycle (−log_10_ (*p* value) = 31.3859) among the biological process category. The most represented GO term was the condensed chromosome kinetochore (−log_10_ (*p* value) = 13.0822) in the cellular component category. Heparin binding (−log_10_ (*p* value) = 6.2714) was the most highly represented term with in the molecular component category. The top ten of the number of deferentially expressed genes and the significance analyzed by GO term are demonstrated in Figure 4.

**Figure 5:**
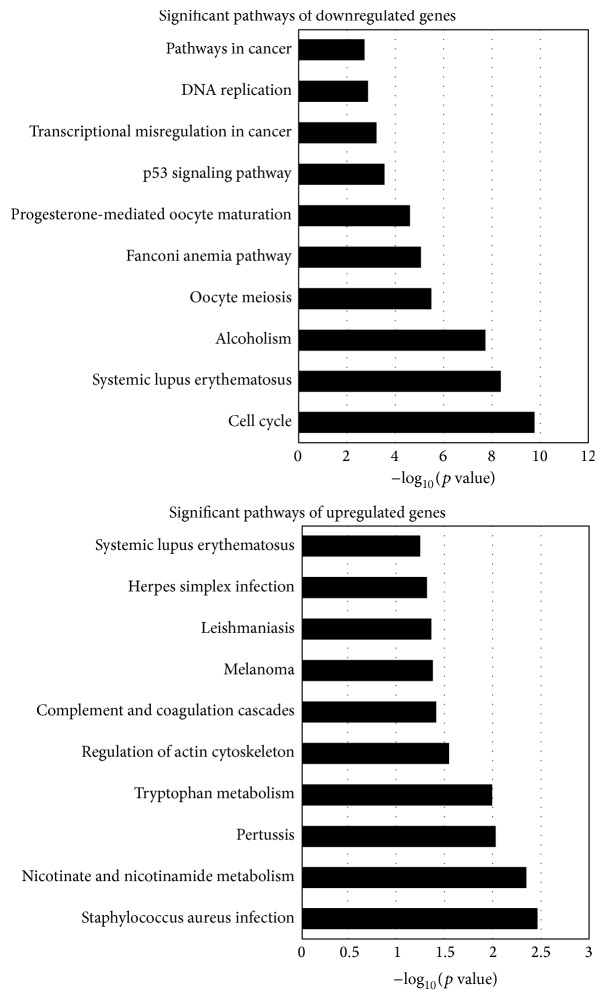
Pathway analysis of the differentially expressed genes. Fisher's exact test was used to select the main pathway, and the significance threshold was defined with* p* value. The lower the* p* value, the more significant the pathway (*p* value ≤0.05 is recommended). We can get some information about the possibility between the differentially expressed genes and the change of cellular pathways.

KEGG (Kyoto Encyclopedia of Genes and Genomes) was used to analyze the pathway enrichment. The pathway analysis showed that changed genes participated in the DNA replication, cell cycle, P53 signaling pathway, pathway in cancer, and melanoma. The upregulated genes participated in 157 pathways while downregulated genes participated in 169 pathways. The top ten pathways among them are shown in Figure 5.

### 3.4. The Regulation of BoNTA on the Expression of FGFR3P and COL19A1

**Figure 6:**
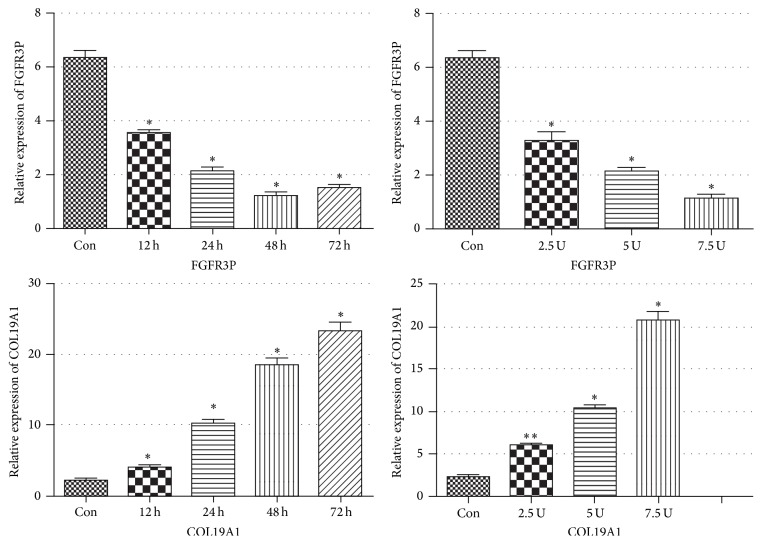
The regulation of BoNTA on the expression of FGFR3P and COL19A1 in HDFs. The expressions of FGFR3P and COL19A1 in HDFs different dosage of BoNTA were measured using qRT-PCR. The heights of the columns in the chart represent the fold changes. Data are the mean ± SEM (*n* = 6). *p* < 0.05 was considered to indicate a statistically significant difference compared with control HDFs. ^*∗*^*p* < 0.05 and ^*∗∗*^*p* < 0.01.

We also performed qRT-PCR to validate the altered lncRNA expression at different time-points and different dosage of BoNTA treatment. We found that the level of FGFR3P reached the lowest at 48 h but indicated an upward trend at 72 h when treated by BoNTA at the dosage of 5 U/10^6^ cells (Figure 6). The level of FGFR3P was gradually increasing with the increasing dosage of BoNTA (at a range from 2.5 U/10^6^ cells to 7.5 U/10^6^ cells). At the same time, the level of COL19A1 in HDFs was gradually increasing with the extension of time treated by BoNTA at the dosage of 5 U/10^6^ cells (Figure 6), and its level showed a trend of increasing when the dosage of BoNTA ranges from 2.5 U/10^6^ cells to 7.5 U/10^6^ cells (Figure 6).

## 4. Discussion

Recently, lncRNAs have come into the extent of probing into human biological functions and diseases [13–17]. Until now, only a few lncRNAs had been reported about HDFs, and studies on the expression of lncRNAs in BoNTA treated HDFs were still lacking. In this study, we identified some differentially expressed lncRNAs and mRNAs between the BoNTA treated group and control group by analyzing gene expression profiles. The number of changed lncRNAs was greater than that of mRNAs. Although currently the precise roles of the changed lncRNAs in BoNTA treated HDFs are not clear, lncRNAs have been regarded as vital regulators of gene expression and have various biofunctions. There are a large number of evidences demonstrating that lncRNAs can regulate gene expression by forming RNA-protein, RNA-RNA, DNA-RNA, and protein-DNA interactions [18]. Although no direct relationship was found between the altered lncRNA and mRNA expressions, we are convinced that the clear changes of lncRNAs (RP11-517C16.2-001, FR271872, LOC283352, RP11-401E9.3, FGFR3P, and XXbac-BPG16N22.5) in HDFs are in response to BoNTA and are related to the changes of protein-coding RNAs. We suspected that the decrease of NOS2 indicated the regulation of cell proliferation process [19, 20]. The increase of FOS induced by BoNTA showed the regulation of proliferation, which is also involved in the cellular senescence process of HDFs [21–24]. BIRC5 has been reported to participate in modulation of diverse cellular processes such as proliferation, adhesion, apoptosis, migration and invasion during growth, development, repair, maintenance, and regression of a wide variety of mesenchymal tissues [25–28]. The downregulated BIRC5 induced by BoNTA indicated the decrease of apoptosis in HDFs. PLAC8 has been proved to regulate cell cycle and participate in the regulation of apoptosis and cell division [29–31].

GO database was applied to analyze the function of the differentially expressed genes. The results were divided into three sections: biological process (BP), cellular component (CC), and molecular function (MF). Besides the top ten of the number of differentially expressed genes and the significance analyzed by GO term demonstrated in Figure 4, the analysis showed that the differentially expressed genes were also involved in a variety of other biological functions, such as negative regulation of autophagy (GO: 0010507), positive regulation of collagen biosynthetic process (GO: 0032967), regulation of G1/S transition of mitotic cell cycle (GO: 2000045), and positive regulation of fibroblast proliferation (GO: 0048147). The analysis indicated that the lncRNAs can affect the function of HDFs by regulating the expression profiles of genes related to HDFs. KEGG analysis can provide some suggestive information about the potential relativity of the changed gene expression with the alterations of the pathways. The result of our study indicates several significant pathways related to a variety of functions, such as cell proliferation, cell cycle, apoptosis, and DNA replication. It had been reported that BoNTA can regulate the process of cell cycle and DNA replication by other researchers. For example, G. Karsenty et al. reported in their study that BoNTA obviously reduced LNCaP cell proliferation and increased apoptosis in a dose-dependent manner [32]. Park et al. found that BoNTA upregulates the expression of cell cycle related genes such as RhoA, Rac1, and Cdc42 in a dose-dependent manner [33]. Our prediction results accord with the functional analysis of BoNTA obtained from other investigations.

QRT-PCR was applied to verify the result of microarray analysis. We learned from other studies that BoNTA can regulate cell proliferation and collagen synthesis; the mechanisms may play important roles in skin rejuvenation effects of BoNTA [32–34]. The RNAs chosen for qRT-PCR confirmation meet at least one of two following criteria: 1: the RNAs being at least 2-fold differently expressed in BoNTA treated groups in comparison with the control group according to the data analysis; 2: the RNAs that had been proved in other studies to have a relationship with biological functions such as cell proliferation and collagen synthesis. The qRT-PCR confirmation results of six lncRNAs (RP11-517C16.2-001, FR271872, LOC283352, RP11-401E9.3, FGFR3P, and XXbac-BPG16N22.5) and mRNAs (NOS2, C13orf15, FOS, FCN2, SPINT1, PLAC8, BIRC5, and COL19A1) were consistent with the microarray data which confirmed the reliability of our microarray analysis.

Next, we applied qRT-PCR to further investigate the expression changes of COL19A1 and FGFR3P after BoNTA treatment at different dosages and culture times. COL19A1, as one member of the fibril-associated collagens with interrupted triple helices (FACIT) group, is thought to act as a cross-bridge between extracellular matrix molecules (ECM) and is involved in the formation of the well-known striated fibrils [35]. Bioinformatic analysis reveals that FGFR3P which is located on chromosome 6 is defined as the pseudogene of Fibroblast Growth Factor Receptor 3 (FGFR3), noncoding RNA. FGFR3 is one member of FGFR family and had been reported to participate in the regulation of cell proliferation, apoptosis, migration, and angiogenesis in many cells including HDFs [36–39]. Although pseudogenes have been considered as the remnants of functional genes which have no coding ability over a long period of time, evergrowing number of studies have proved that some pseudogenes have diverse functions, including serving as miRNA decoys, functioning as antisense transcripts, encoding short peptides, and producing siRNAs or proteins [40–43]. Our results showed that downregulating effect of BoNTA on the expression of FGFR3P was time-related. Meanwhile, the dosage of BoNTA at a range from 2.5 U/10^6^ cells to 7.5 U/10^6^ cells increased the expression of FGFR3P and COL19A1 in HDFs as well. We speculated significant change of expression of these RNAs in a time-dependent manner after BoNTA treatment which may be of some clinical significance. Zhu et al. conducted one clinical experiment and found that topical application of BoNTA could enhance the rejuvenation effect of fractional CO_2_ laser; they also found that best results of skin detection were in the last observation point at 3 months after treatment [34]. Zhu et al. also performed another clinical study and found that intradermal BoNTA injection showed its best rejuvenation results in the last observation point at 12 weeks after treatment as well [44]. The results of the previous clinical investigations showed that BoNTA had a persistent promoting effect of collagen synthesis with the extending of time. Taking these into consideration, we speculate that BoNTA have the antiaging effect not only by relaxing in muscle fiber, but also by promoting activity of HDFs directly. The underlying mechanisms still need further studies.

In conclusion, for the first time, our current study identified the changes of expression profiles of lncRNAs in BoNTA treated HDFs and found that BoNTA dynamically regulated the expression of COL19A1 and FGFR3P in HDFs, indicating the potential role of several lncRNAs in BoNTA treated HDFs. Therefore, further explorations are warranted to discover the mechanisms behind the dynamic changes BoNTA induced on COL19A1 and FGFR3P and the findings obtained in this study should lay foundations for further studies into the potential roles of these altered lncRNAs in BoNTA treated HDFs.

## Figures and Tables

**Table 1:** Primer sequences for long noncoding RNAs and protein-coding RNAs.

Gene name	Primer sequences
RP11-517C16.2-001	Forward primer: TTTGTCAACGGGCTCTACCC
	Reverse primer: TTTGTCAACGGGCTCTACCC

FR271872	Forward primer: CACCTCCTTCCCTGGACTAGA
	Reverse primer: CACCTCCTTCCCTGGACTAGA

LOC283352	Forward primer: AAGGGTTTATGTGCTCGGAGG
	Reverse primer: CTGGCTGAGGAGTCTCACTT

RP11-401E9.3	Forward primer: CCAGTCATGCCCATCCAGAA
	Reverse primer: CCATGCAGCAACTAGCAAAGG

FGFR3P	Forward primer: ATGGAAGGCTGCTTCATGCT
	Reverse primer: GTTTCAAGACCTCAGCGGGA

XXbac-BPG16N22.5	Forward primer: AGACTCAAGGGGACCAGACC
	Reverse primer: CTGCAGGCAGGTGTATCTCA

COL19A1	Forward primer: TCGAGTACGAAGAAACGCCAA
	Reverse primer: TGCCACTGACGATCAAACAAA

NOS2	Forward primer: ACATCGACCCGTCCACAGTAT
	Reverse primer: CAGAGGGGTAGGCTTGTCTC

C13orf15	Forward primer: CTGAATTCTCCAACAGACT
	Reverse primer: ATGGGAAAGCTTACTGCT

E2F1	Forward primer: AGCTGGACCACCTGATGAAT
	Reverse primer: GAGGGGCTTTGATCACCATA

SPINT1	Forward primer: AGACTACTGCCTCGCATCCAA
	Reverse primer: CAAGCAGCCTCCATAAACGAA

FOS	Forward primer: TGACAGATACACTCCAAGCGG
	Reverse primer: GGGAAGCCAAGGTCATCG

FCN2	Forward primer: GTAAAACGACGGCCAGTTATGGCCCTGCTTCTTCCTC
	Reverse primer: TTCCAGAGTGTGTTCTCCCAC

PLAC8	Forward primer: CCTCTACACTGCCTCAGCATC
	Reverse primer: GTAAAACGACGGCCAGTTTCTACACAATAAGGGAGGAATGG

BIRC5	Forward primer: AGGACCACCGCATCTCTACAT
	Reverse primer: AAGTCTGGCTCGTTCTCAGTG

**Table 2:** Differentially expressed long noncoding RNAs in HDFs (fold change > 5).

Probe name	Gene symbol	Expression	Chromosome	Strand	Start	End	Fold change
CUST_18266	RP11-262H14.3-005	Up	Chr9	−	66513657	66553531	18.14
CUST_28663	linc-TCTE3-3	Up	Chr6	−	170470884	170475667	9.33
CUST_73981	linc-GRAMD3-2	Up	Chr5	+	124372380	124486527	8.12
CUST_25057	Z83001.1-003	Up	Chr11	−	31710730	31789373	7.07
CUST_54220	RP11-517C16.2-001	Up	Chr16	−	84492865	84500967	6.13
CUST_77914_PI429545380	RP11-444D13.1-001	Up	chr1	−	183723553	183724072	5.70
CUST_42824_PI429545402	RPS2P41	Up	chr12	−	112317141	112318053	5.63
CUST_24581_PI429545380	FR271872	Up	chr1	+	40364066	40364332	5.55
CUST_27762_PI429545402	KLRD1	Up	chr12	+	10378664	10467608	5.45
CUST_42463_PI429545376	linc-SCGB1D4-2	Up	chr11	−	62178649	62179162	5.35
CUST_83283_PI429545395	RSPH10B	Up	chr7	−	5995734	6002990	5.34
CUST_63264_PI429545410	LOC283352	Up	chr12	+	129594234	129597843	5.34
CUST_70508_PI429545399	RP11-401E9.3	Up	chr10	−	7875580	7875920	5.05
CUST_70526	MTHFD1	Down	Chr14	+	64924714	64926721	−25.69
CUST_86832	AC010136.2-001	Down	Chr2	+	218843430	218857338	−8.43
CUST_75452	PNKP	Down	Chr19	−	50369397	50370818	−8.04
CUST_89880	STPG1	Down	Chr1	−	24717747	24742643	−7.77
CUST_45703	RP11-643A5.2-002	Down	Chr15	−	54239821	54267147	−6.66
CUST_15063	FGFR3P	Down	chr6_cox_hap2	+	2857852	2858455	−6.52
CUST_29613	linc-HOXA11	Down	Chr7	−	27226865	27232305	−6.25
CUST_33771	FR081392	Down	Chr6	+	161943497	161943737	−6.21
CUST_75093	CTA-292E10.6-001	Down	Chr22	+	29196671	29244547	−6.04
CUST_90031_PI429545395	RP11-73B2.7	Down	chr7	−	63398282	63398887	−5.63
CUST_81910_PI429545380	AC007131.2-003	Down	chr2	−	59465851	59476702	−5.5
CUST_92526_PI429545402	ZFAND6	Down	chr15	+	80364932	80413144	−5.2
CUST_62442_PI429545395	XXbac-BPG16N22.5	Down	chr6	+	31483755	31483988	−5.14
CUST_19370_PI429545380	FR316649	Down	chr3	+	188550879	188551155	−5.07

**Table 3:** Differentially expressed protein-coding RNAs in HDFs (fold change > 3).

Probe name	Gene symbol	Expression	Chromosome	Strand	Start	End	Fold change
A_33_P3367396	FAM177B	Up	Chr1	+	222923479	222923538	30.05
A_33_P3400699	SLC26A5	Up	Chr9	−	10 2993259	102993200	5.16
A_23_P502464	NOS2	Up	Chr17	−	26083921	26083862	4.21
A_33_P3423270	TMEM40	Up	Chr3	−	12775584	12775525	4.08
A_24_P183128	PLAC8	Up	Chr4	−	84015842	84012077	4.03
A_23_P135226	OR1N2	Up	Chr9	+	125316281	125316340	3.91
A_23_P153616	MADCAM1	Up	Chr19	+	505192	505251	3.89
A_23_P49060	SPINT1	Up	Chr15	+	41149316	41149375	3.58
A_24_P10137	C13orf15	Up	Chr13	+	42042937	42044635	3.42
A_24_P630490	DFNB59	Up	Chr2	+	179325165	179325759	3.38
A_23_P106194	FOS	Up	Chr14	+	75748214	75748273	3.05
A_23_P313588	TMPRSS6	Up	Chr22	−	37480125	37480066	3.04
A_23_P134085	CNKSR3	Down	Chr6	−	154726608	154726549	−14.99
A_24_P323598	ESCO2	Down	Chr8	+	27662101	27662160	−5.44
A_23_P216756	FCN2	Down	Chr9	+	137779164	137779223	−4.27
A_24_P225616	RRM2	Down	Chr2	+	10270487	10270546	−4.19
A_23_P15844	BRIP1	Down	Chr17	−	59760967	59760908	−4.04
A_23_P126212	CLSPN	Down	Chr1	−	36204176	36204117	−3.85
A_33_P3807062	HJURP	Down	Chr2	−	234746088	234746029	−3.84
A_23_P100127	CASC5	Down	Chr15	+	40917525	40917584	−3.81
A_33_P3257678	HIST2H3A	Down	Chr1	+	149824626	149824685	−3.79
A_33_P3326210	ESCO2	Down	Chr8	+	27660830	27660889	−3.72
A_23_P8452	LFNG	Down	Chr7	+	2567971	2568030	−3.70
A_23_P51085	SPC25	Down	Chr2	−	169728015	169727956	−3.61
A_23_P130182	AURKB	Down	Chr17	−	8110917	8110655	−3.59
A_23_P254733	MLF1IP	Down	Chr4	−	185616396	185616337	−3.49
A_23_P133956	KIFC1	Down	Chr6	+	33374428	33374621	−3.48
A_24_P214231	STIL	Down	Chr1	−	47716850	47716791	−3.45
A_23_P131330	LRRTM1	Down	Chr2	−	80529291	80529232	−3.45
A_32_P96719	HIST1H2AL	Down	Chr6	−	27833502	27833561	−3.27
A_23_P363174	GTSE1	Down	Chr22	+	46725391	46725450	−3.23
A_23_P118246	GINS2	Down	Chr16	−	85711713	85711654	−3.20
A_24_P322354	SKA1	Down	Chr18	+	47919899	47919958	−3.14
A_23_P118815	BIRC5	Down	Chr17	+	76220720	76220779	−3.08
A_24_P413884	CENPA	Down	Chr2	+	27016914	27016973	−3.06
A_24_P314571	SPC24	Down	Chr19	−	11257053	11256994	−3.05
A_23_P99292	RAD51AP1	Down	Chr12	+	4668182	4668241	−3.01
A_23_P80032	E2F1	Down	Chr20	−	32264048	32263989	−3.01
