# Corrigendum to “The Effect of Botulinum Toxin Type A on Expression Profiling of Long Noncoding RNAs in Human Dermal Fibroblasts”

**DOI:** 10.1155/2019/1594837

**Published:** 2019-11-26

**Authors:** Ying-Ying Miao, Juan Liu, Jie Zhu, Yan-Ling Tao, Jia-An Zhang, Dan Luo, Bing-Rong Zhou

**Affiliations:** ^1^Department of Dermatology, The First Affiliated Hospital of Nanjing Medical University, Nanjing 210029, China; ^2^Department of Dermatology, The First Affiliated Hospital of Nanjing University of TCM, Nanjing, Jiangsu 210029, China

In the article titled “The Effect of Botulinum Toxin Type A on Expression Profiling of Long Noncoding RNAs in Human Dermal Fibroblasts” [1], there was an error in Figure 4, where the second and third graphs of Figure 4(a) were mistakenly duplicated during the production process. The correct figure is shown below.

## Figures and Tables

**Figure 4:**
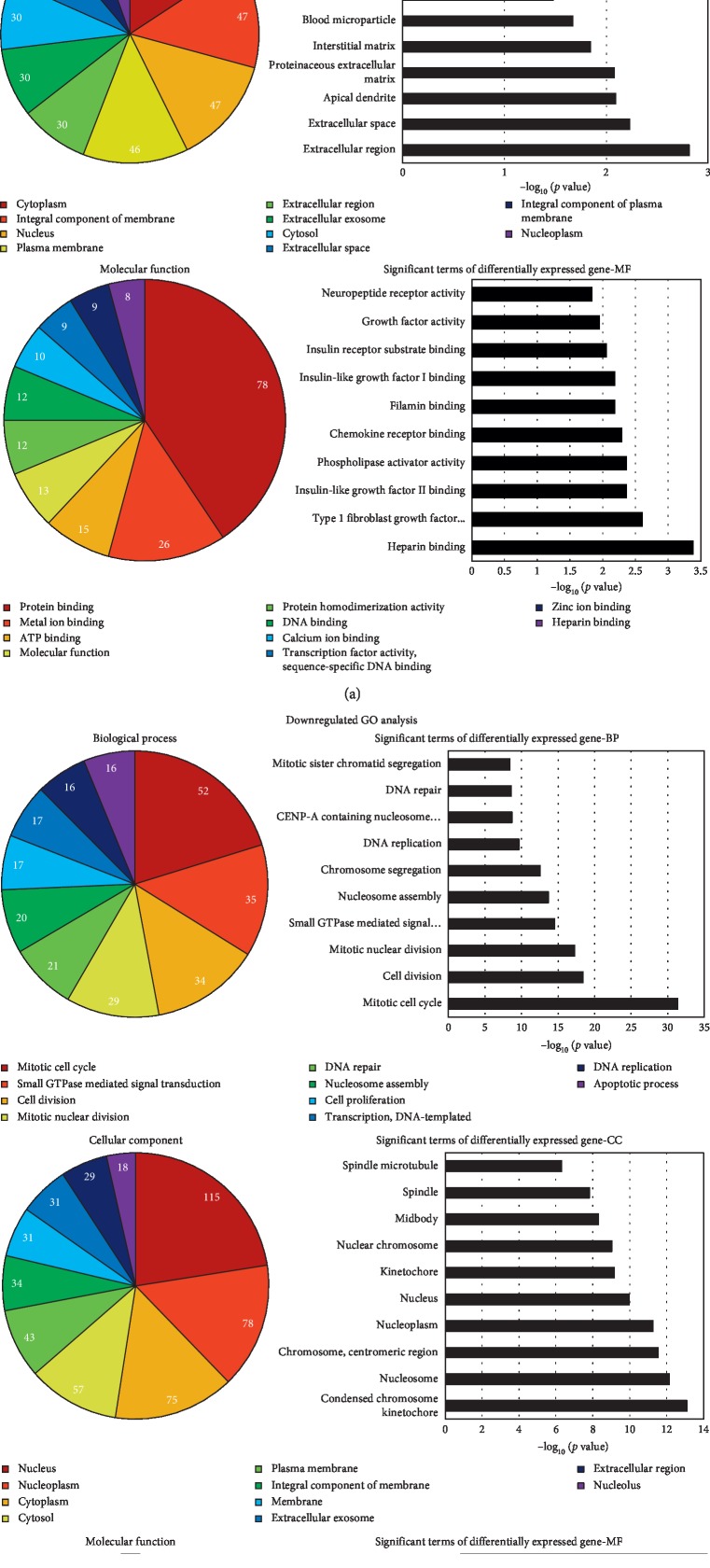
Bioinformatic analysis of the differentially expressed genes. The *p* value denotes the significance of GO terms enrichment in the differentially expressed genes. The lower the *p* value, the more significant the GO term (*p* value ≤0.05 is recommended). We can choose the target genes for further study based on the combination of the analysis provided by GO and the biologic significance.
